# Analyzing the effect of health reforms on the efficiency of Ecuadorian public hospitals

**DOI:** 10.1007/s10754-023-09346-z

**Published:** 2023-03-16

**Authors:** Juan Piedra-Peña, Diego Prior

**Affiliations:** 1 Department of Applied Economics, Universitat Autònoma de Barcelona, Campus Bellaterra, Barcelona, Spain; 2 Department of Business, Universitat Autònoma de Barcelona, Campus Bellaterra, Barcelona, Spain

**Keywords:** Healthcare efficiency, Metafrontier, Health reforms, Panel data DEA, I18, C14, H51

## Abstract

This study aims to assess whether Ecuadorian health reforms carried out since 2008 have affected the efficiency performance of public hospitals in the country. We contribute to the literature by shedding new light on the effects on public healthcare efficiency for developing countries when policies move toward health equity and universal coverage. We follow a two-stage approach, wherein the first stage we make use of factor and cluster analysis to obtain three clusters of public hospitals based on their technological endowment; we exploit Data Envelopment Analysis for panel data in the second stage to estimate robust efficiency measures over time. Our innovative empirical strategy considers the heterogeneity of healthcare institutions in the analysis of their efficiency performance. The results show a significant decrease in the average efficiency of low and intermediate technology hospitals after the new constitution was adopted in 2008. The decline in efficiency coincides with the two reforms of 2010 and 2011 that brought on higher social security coverage.

**Supplementary Information:**

The online version contains supplementary material available at 10.1007/s10754-023-09346-z.

## Introduction

As a determinant of population wellbeing and economic growth, improving health has become a major topic in economic debates and features high on the public policy agenda in many countries around the world. Healthcare is one of the main public policies implemented by most governments and improving the efficiency of its delivery is a crucial goal of health service providers globally (Au et al., 2014). Nevertheless, health systems in Latin America face specific challenges, including insufficient human resources and training, lack of evaluations of strategy outcomes, operating levels, and weaknesses in the public health system’s response capacity, among others (Ruiz-Rodriguez et al., 2016).

In this context, the Ecuadorian health sector has undergone a continuous process of deterioration as a consequence of neoliberal reforms carried out in the 1990 s (Homedes and Ugalde, 2005) and the crisis of 2000, which mainly affected the most deprived population. This deterioration meant a progressive reduction of the health budget, lack of infrastructure investment and shrinking human resources, and low quality and coverage of public services (López-Cevallos & Chi, 2010).

In 2008, the government of former President Rafael Correa brought in a new constitution that guarantees health as a citizens’ right and introduced a series of health reforms that moved toward universal coverage and free primary medical services. This change was accompanied by substantial public investment in the health sector in order to improve the quality and quantity of medical services (De Paepe et al., 2012; Hartmann, 2016).

There are two potential effects of these reforms on the efficient delivery of medical services to the population. On the one hand, the increased demand for health services by the newly insured population might encourage hospitals that were not using their spare capacity and/or medical resources correctly to take full advantage of them by optimizing their resources and delivering a more efficient service. On the other hand, in the desire to promote equal access to health, these policies might lead to over-demand for health services that hospitals are unable to cope with in the short term (Smith & Yip, 2016).

It is clear that improving the efficiency of resource use is a key issue in most health systems, and is particularly acute in developing countries where there is a pressing need for proper resource allocation given the limited level of overall infrastructure, resources and health budget (Hafidz et al., 2018).

In light of the above, a relevant question arises: when the objective of equity provides the rationale for governments’ central involvement in healthcare, is healthcare efficiency affected? We contribute to this topic by focusing on the Ecuadorian context, which offers a framework of analysis characterized by health reforms designed to bring in universal coverage and seeking the “well-living” of the population (Espinosa et al., 2017). We present an analysis with current information on the efficiency changes in a reality that is still adapting to these reforms, and must face potential problems arising in the short term. Thus, this study aims to tackle two objectives. First, we estimate the technical efficiency of public hospitals over time, considering the period from 2006 (starting before the new government came to power) until 2014. Then, we consider the public health reforms introduced since 2008 in order to assess whether they have affected the efficiency performance of public hospitals in Ecuador.

To properly account for the situation in Ecuador, we need to consider its existing regional disparities, deeply exacerbated by the neoliberal reforms carried out in the 1990 s (Hartmann, 2016). One outcome of this process is the marked technological heterogeneity among its health institutions, with a greater concentration of hospitals with higher levels of technology in the most developed regions.

In order to consider the aforementioned technological differences in the Ecuadorian public health system, we introduce a methodological innovation in a two-stage analysis. In the first stage, we use multivariate factor analysis and clustering techniques to find homogeneous groups with uncorrelated characteristics of technological endowment. We compare them with a common frontier, similarly to the metafrontier approach (Battese and Rao, 2002; Battese et al., 2004; O’Donnell et al., 2008) but with some modifications that need to be carried out to control for the lack of data and the system’s heterogeneity. We propose an alternative approach, based on the seminal studies of Banker and Morey (1986, 1996) and Podinovski (2005).

Having identified these new clusters, in the second stage we combine the metafrontier and panel data DEA (Surroca et al., 2016; Pérez-López et al., 2018) to account for robust efficiency values over time. Considering an empirical methodology that allows for this heterogeneity enables us to obtain consistent efficiency values, which might otherwise be biased if we applied classical efficiency measurement techniques (like DEA or Malmquist index) to the whole sample (Mitropoulos et al., 2015).

The paper begins with a brief contextualization of the Ecuadorian healthcare system, in Sect. 2. Section 3 then explains and reviews the methodological framework and the most recent cited empirical literature; the methodology is presented in Sect. 4. In Sects. 5 and 6 we discuss the data and results obtained. Finally, in Sect. 7 we present the main conclusions.

## Institutional context

Ecuador’s healthcare system combines the public and private sectors. The public sector comprises the Public Ministry of Health (MSP), the Ministry of Social and Economic Inclusion (MIES), the municipal health services and the social security institutions.^1^ The MSP provides health services to the whole population, the MIES and municipality health programs supply medical care to those without insurance, while the social security institutions cover the affiliated working population (Lucio et al., 2011). Since Rafael Correa’s government came to power in 2007 and implemented the new constitution in 2008, an unprecedented level of public investment has taken place in Ecuador, focusing on primary services such as education and health. This new government, called the "Citizen Revolution", marked the beginning of a stage of democratic stability that gave the State the central role that guarantees and promotes the enjoyment of rights for the entire population.

Additionally, the 2008 constitution also brought in significant changes, especially in access to health services and social security coverage. Articles 3 and 34 of the National Constitution state that health is a right guaranteed by the State and it shall ensure the full exercise of the right to social security. On this basis, several reforms have been implemented such as coverage of children under the age of 18 in 2010 (Article 102, Social Security Law) or deprivation of liberty for employers who do not affiliate workers within a maximum period of 30 days in 2011 (Art. 244, Organic Comprehensive Criminal Code), resulting in a significant increase in the number of active beneficiaries until 2014 (Orellana et al., 2017).

**Fig. 1:**
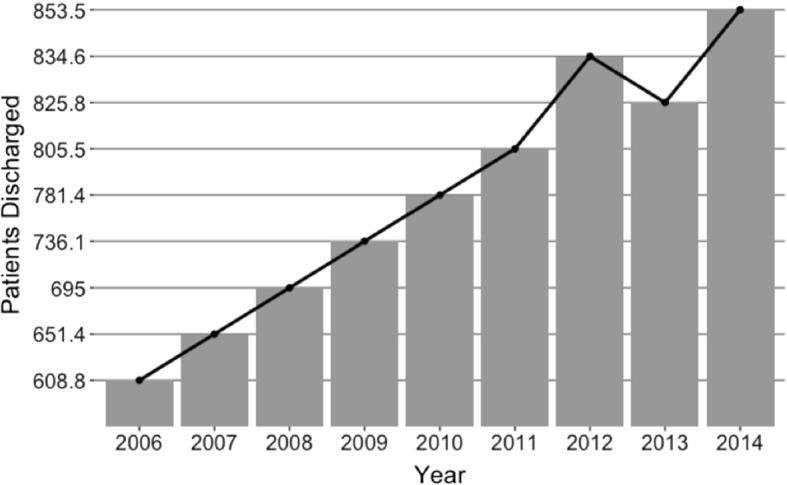
Number of discharged patients in public hospitals in the period 2006–2014 (in thousands)

Some results can be drawn from the Annual Survey of Hospital Beds and Discharges and the Survey of Health Activities and Resources. Figure 1 shows that between 2006 and 2014 the total number of patients discharged from public hospitals rose from 608 thousand to over 853 thousand, representing a 40 percent increase in patients attended. The biggest jump in medical attention was seen in 2012, which coincides with the period following the above-mentioned reform of 2011.

**Fig. 2:**
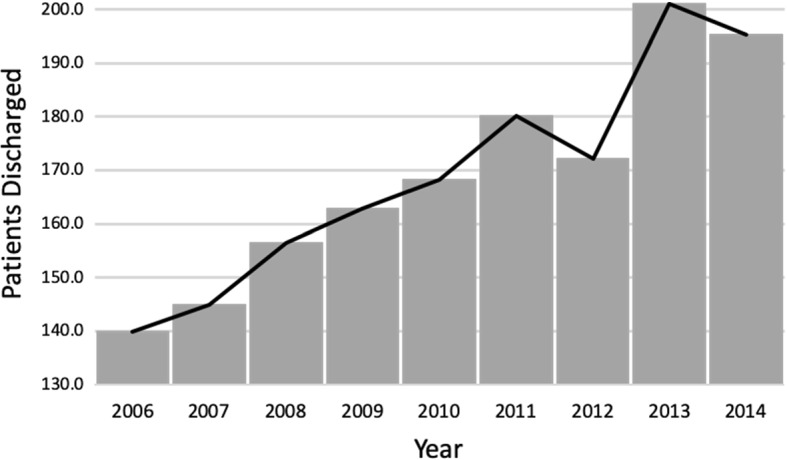
Number of discharged patients from private clinics in Quito, Guayaquil and Cuenca, 2006–2014 (in thousands)

**Fig. 3:**
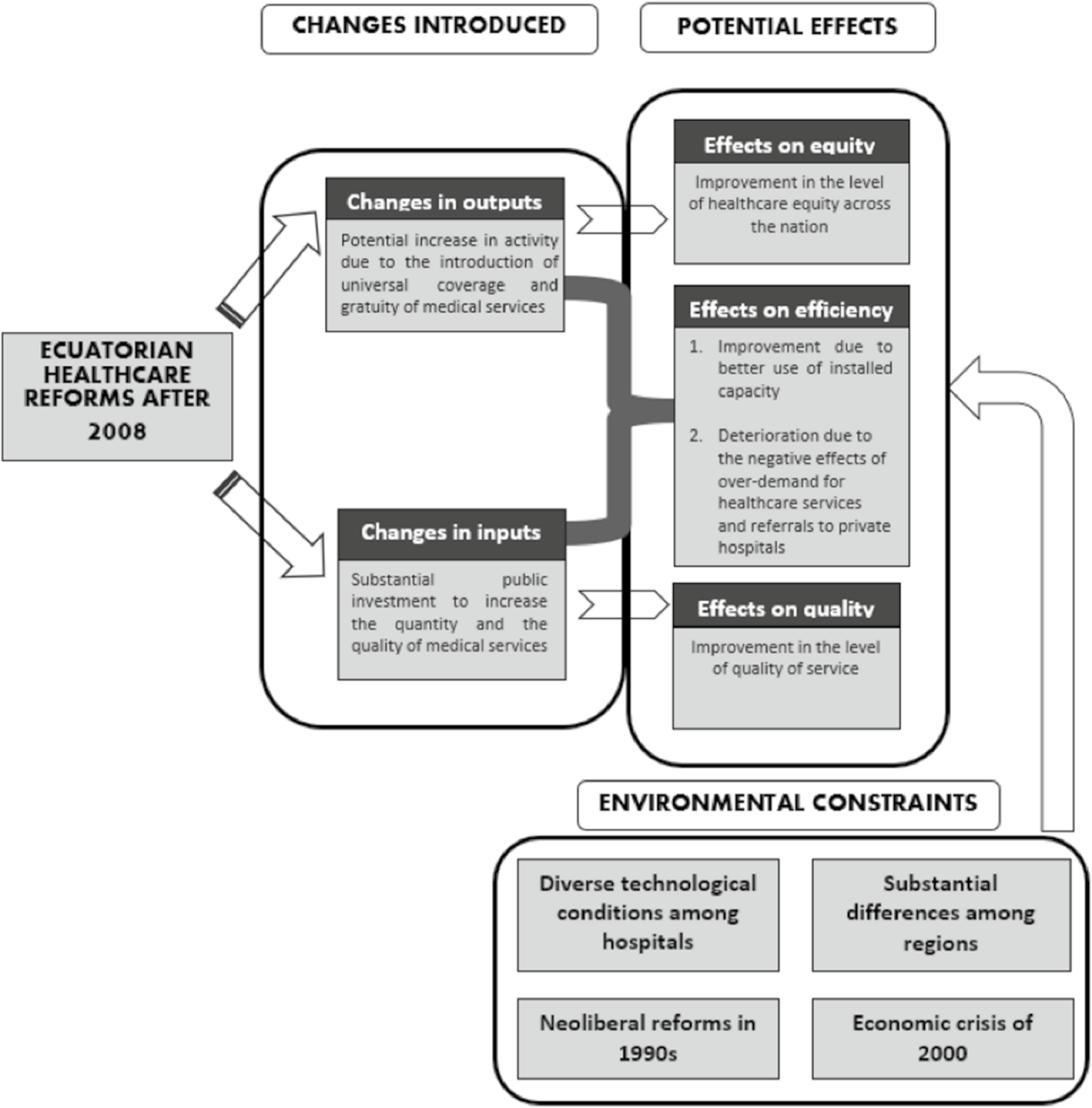
Dynamic in the Ecuadorian healthcare system

The final goal of these reforms was to improve the wellbeing of the most deprived citizens, in pursuit of a more equal access to medical services. However, despite the new investment in infrastructure and human capital, little attention was paid to how these new health improvements would affect the performance of the hospitals. De Paepe et al. (2012), discusses how the introduction of these new free services and the increase in the insured population brought about a “demand crisis”, especially in larger cities. This higher demand meant that public hospitals could not cope with the influx of patients and drove the need to contract private services to stem public discontent. Some evidence of this measure can be found in Fig. 2, where 2011 and 2013 present the biggest jump in patients attended in private clinics for three of Ecuador’s largest and most densely populated cities (Quito, Guayaquil and Cuenca). Unfortunately we do not have information on the patient referrals to private healthcare institutions, but the decrease in discharged patients from public hospitals in 2013 (Fig. 1) might be signaling a small alleviation in the demand for public healthcare services that seems to have been referred to private clinics (Fig. 2). The dynamic deriving from the above-mentioned changes introduced in the healthcare system is illustrated in Fig. 3.

These facts reveal the need for an empirical strategy with which to measure the efficiency changes and the potential effects of the public health reforms. The following section reviews the methodological framework most commonly used in the literature to address this relationship.

## Literature review

This paper takes its methodological framework from Production Theory (Debreu, 1951; Koopmans, 1951; Farrell, 1957) and the metafrontier production (Battese et al., 2004; O’Donnell et al., 2008). The main idea of productive efficiency is linked to the concept of Pareto Efficiency Allocation, according to which a resource endowment is efficient when there is no other possible allocation that makes a Decision Making Unit (DMU) better off.^2^ The efficiency analysis can be applied to any DMU, and we can distinguish between technical efficiency and allocative efficiency. The latter assumes that relative prices are known and are reasonably stable. Following Farrell (1957), an efficient unit would obtain a value of one, and it could take an input or an output orientation. The former focuses on minimizing the input use and the latter on maximizing the output obtained in the production process.

In turn, the metafrontier production assumes that DMUs from different environmental conditions, regions, and/or countries face different production opportunities and have to make different choices taking into account variations in the feasibility of input–output combinations. These *technology sets* will therefore be different and difficult to compare. Battese et al. (2004) and O’Donnell et al. (2008) develop a way to make efficiency comparisons across groups of DMUs. They do this by measuring efficiency relative to a common *metafrontier* which is defined as a boundary of an unrestricted technology set, and they also define group frontiers to be boundaries of limited technology sets that are embedded in the common frontier.

The metafrontier envelops the group frontiers. Efficiencies that are measured with respect to the metafrontier can be decomposed into two components: one component measures the distance from an input–output vector to the group frontier, which is the common measure of technical efficiency; and a second component measures the distance between the group frontier and the metafrontier, which is defined as a *technological gap ratio* (TGR), and represents the restrictive nature of the production frontier.

In the empirical literature, healthcare efficiency measurement has attracted growing interest over the years. Most studies focus on measuring the efficiency and productivity of healthcare using parametric and non-parametric applications. Several authors offer extensive reviews of the published literature (Hollingsworth, 2003, 2008; Worthington, 2004; O’Neill et al., 2008; Cantor and Poh, 2018). However, more than half of these were applied in the US and Europe, while just a few have examined developing countries, although this number has been rapidly increasing over the last years (Hollingsworth, 2008).

There is an extensive literature that applies parametric techniques to measure healthcare efficiency. Parametric techniques, in general, are regression-based and assume a specific functional form from the frontier. In this line, stochastic frontier analysis (SFA) has been used as the main parametric tool for efficiency measurement. Unlike ordinary least squares (OLS), it models the error term in two parts: one that measures the distance from the frontier (inefficiency), and the other measuring the statistical noise (O’Neill et al., 2008). In the healthcare applied literature, to measure its evolution over time, it is common to use panel models assuming either Cobb-Douglas (Varabyova & Schreyögg, 2013), the translog functional form (Ferrari, 2006; Rosko, 2001), or both (Herr, 2008; Herr et al., 2011). Other approaches like Hamidi (2016) perform a comparative application using three functional forms: Cobb-Douglas, translog and multi-output distance functions. Being the former the one that better fits their data. However, in our application the multidimensional nature of public hospitals, with different functions that are difficult to quantify, plus the impossibility to obtain input and output prices information makes the assumption of a functional form difficult to defend. In this spirit, technical efficiency can be very sensitive to the choice of a functional specification (Giannakas et al., 2003), which lead us to rely in non-parametric approaches that do not need to assume a production function a priori.

Here, data envelopment analysis (DEA) has excelled over other techniques, as a non-parametric linear programming method for measuring relative efficiency of homogeneous DMUs. This approach is more consistent with economic theory as it locates technical or Pareto inefficiencies instead of measuring efficiency based on averages (O’Neill et al., 2008; Cantor and Poh, 2018). It also allows a data driven assessment of the production process without strong assumptions about the functional form, which is a major advantage in the face of uncertainty (Staat, 2011).

In this regard, the published literature applied to Latin American countries has been somewhat scarce. De Castro et al. (2010a) use network DEA to assess the performance and integration of healthcare and teaching dimensions in Brazilian university hospitals. Keith and Prior (2014) measure technical efficiency using the DEA approach and evaluate the potential presence of scale and scope economies in Mexican private medical units. Ruiz-Rodriguez et al. (2016) also apply DEA analysis in a four-stage approach along with a series of Tobit regressions in order to estimate the technical efficiency of the three women’s health promotion and disease prevention programs in Bucaramanga, Colombia. However, these studies aim to measure efficiency in a specific year analysis, and none of them attempted to identify the effects of health reforms.

Following this line, several authors have addressed research questions regarding the relationship between health reforms and performance (e.g. Linna 1998, Maniadakis et al., 1999, Van Ineveld et al., 2016), but very few have focused on Latin American countries (Arocena and García-Prado, 2007; De Castro et al., 2010b). Most of them make use of non-parametric methods, like DEA models, to calculate efficiency scores and Malmquist productivity indices, subsequently decomposable on efficiency and technological change, which have been widely employed in the literature in part because they require neither relative price information nor restrictive behavioral assumptions for their estimation (Chowdhury et al., 2014).

Table 1 presents a summary of the most recent cited literature on health reforms and hospital performance using—mostly-non-parametric models. To answer questions regarding health reforms, the literature has mainly followed two approaches that rely largely on the availability of the data, and the results may depend on the context in which it took place and the type of reform implemented.

**Table 1:** Summary of the literature

Authors	Country	Year of the reform	Study period	Methodology	Conclusions
Linna (1998)	Finland	1993	1988–1994	Time-varying SFA, DEA, Malmquist index	3–5% annual average increase in productivity due to cost efficiency and technological change. The state subsidy reform of 1993 did not seem to have any observable effects on hospital efficiency
Maniadakis et al. (1999)	Scotland	1990	1991–1996	Malmquist index	Overall net gain in productivity, although a slowdown was observed in the first year after the reform. The change was due to technological change as hospitals were relatively efficient at the time of the reform
Sommersguter-Reichmann (2000)	Austria	1997	1994–1998	DEA, Malmquist index	Positive shift in technology between 1996 and 1998 as a result of the financing reform
Arocena and García-Prado (2007)	Costa Rica	2000	1997–2001	Generalized distance functions, Malmquist index	Improvement in hospital performance mainly driven by quality changes and particularly significant for small hospitals. Productivity growth is mainly due to technical and scale efficiency rather than technological change
De Castro et al. (2010b)	Brazil	2004	2003–2006	DEA, Malmquist index	Financial reform was a good stimulus for efficiency gains, but technology change was not able to take place
Van Ineveld et al. (2016)	The Netherlands	2005	2005–2010	DEA, Malmquist index	Larger differences in efficiency among hospitals. In 2009–2010 the number of larger and more efficient hospitals decreased
Valdmanis et al. (2017)	Scotland	Series of reforms through the period of analysis	2003–2007	Malmquist index, time-series trend analysis	No steady movement with the use of the Malmquist index, but the time-series trend analysis revealed a trend of improvement in technological but not technical change
Xenos et al. (2017)	Greece	2008	2009–2012	DEA, Malmquist index	Negative impact of the crisis in 2009 with 91% of the hospitals achieving a score lower than one. Improvement between 2010 and 2011 followed by stabilization in 2011–2012
Giménez et al. (2019)	Colombia	1993	2009–2013	Mamlquist–Luenberger index	Total productivity worsened by 1% during the period of analysis, mainly due to technological backlash

On the one hand, the hospitals’ performance can be evaluated by considering their performance after a certain reform has taken place. For example, Maniadakis et al. (1999) use Malmquist indices of productivity and quality to evaluate the reforms of the UK National Health Service in the early 1990 s in acute Scottish hospitals over the first 5 years of the reforms. Overall, they find that the hospitals showed a gain in productivity, although an initial regress was observed in the first year after the reform. The changes in productivity were led by technological rather than efficiency changes, given that hospitals were operating close to the industry boundary at the time of the reform and their position changed little over time. Van Ineveld et al. (2016) assess the productivity performance of Dutch hospitals since the health system reform of 2005. They use DEA based measures in a cross-sectional and longitudinal analysis as well as the Malmquist index; they find that the efficiency gap among hospitals has widened, benefiting some of the smaller hospitals but not some larger ones, which might be a consequence of the 2005 reform. Xenos et al. (2017) study the dynamics of efficiency and productivity in Greek public hospitals after the 2008 financial crisis, where in the period of study (2009–2012) hospital budgets were reduced by 40%. Using DEA and bootstrapping Malmquist analysis they find a negative impact in productivity due to the crisis in 2009 with a recovery in 2010 and a posterior stabilization. The latest study conducted in Latin America is that of Giménez et al. (2019). They analyzed the performance of level 1 Colombian hospitals for the period 2009–2013 to evaluate how the health system was performing after the 1993 reform. They also extended the analysis to find out whether the efficiency of high-level hospitals was affected by patient referrals from primary care centers. Using the Malmquist–Luenberger index, they found that productivity decreased by 1% during the period of analysis, providing evidence of a deficient performance in public hospital efficiency after the 1993 reform.

On the other hand, the approach can use a before-after design, which can further benefit the analysis, given that we can actually check the hospitals’ behavior after the reform and gain an initial insight into its influence on their performance. Linna (1998) study the development of cost efficiency and productivity of Finnish hospitals before and after the 1993 state subsidy reform. This author uses panel data Stochastic Cost Frontier models as well as DEA and the Malmquist productivity index and finds that productivity progress was due to both technological change and cost efficiency change; however the state subsidy reform did not seem to have any observable effects on hospital efficiency since it appears to have been improving well before the reform. Sommersguter-Reichmann (2000) studies the 1997 hospital financing reform in Austria and evaluates the changes in productivity between 1994 and 1998. Using DEA and the Malmquist index, she finds a technological improvement was an immediate consequence of the financing reform. Arocena and García-Prado (2007) analyze how Costa Rican hospital efficiency and quality responded to the reforms carried out over the period 1997–2001. They use a generalized output distance function to obtain a Malmquist index that accounts for productivity changes while controlling for quality of care, and find an overall improvement in hospital performance following the reforms due to an increase in quality rather than a better use of resources, and more notably for small hospitals. De Castro et al. (2010b) evaluate the performance and productivity changes for Brazilian Federal University hospitals before and after the financing reform of 2004. Using DEA and the Malmquist index, they find that the financial reform gave the hospitals an opportunity to gain efficiency, but not for technological change. Valdmanis et al. (2017) applied the Malmquist index and time-series trend analysis to assess the shift in efficiency and technology in Scottish hospitals over the period 2003–2007 where health reforms required them to improve their services with fixed budget constraints. They did not find a consistent direction of either improvement or devolution; however, through the use of time-series analysis, they found a trend of growth in technological change.

To the best of our knowledge, most of the literature has relied on a Malmquist index analysis along with other parametrical and non-parametrical approaches. However, none of them have tried to account for technological heterogeneity that may arise when studying hospital performance; just a few studies have considered, at most, hospital size. In this sense, while the technical efficiencies of DMUs measured with respect to a given frontier are comparable, some problems might arise among hospitals that operate under different technologies (Mitropoulos et al., 2015). The efficiency of hospitals that work under a specific production technology cannot be comparable with those of different technology. This problem might be minimized in a context where the country is relatively centralized, and there are no significant regional differences; this might reinforce the homogeneity of the sample (Arocena and García-Prado, 2007). However, in a country like Ecuador, where regional heterogeneities have proved to be strong drivers of the socio-economic reality, the need to account for the potential heterogeneities is crucial.

Recent papers like Mitropoulos et al. (2015) and Chen et al. (2016) have tried to account for some technological differences in the health sector, but their grouping criterion is somewhat subjective and–in the first case–focused on a cross-sectional study. The need to find a clear criterion to group the hospitals in our sample has an important relevance. There is no clearly established way to separate them into homogeneous groups. A priori, the units can be grouped on the basis of geographical, economic or political boundaries.

Here, our aim is to take into account technological heterogeneities among the hospitals by considering their resources and capacity. To this end we consider cluster analysis. O’Donnell et al. (2008) encouraged the use of multivariate techniques when natural boundaries are unavailable. This method has been previously adopted by Balaguer-Coll et al. (2013) to assess the provision of public services and facilities in Spanish municipalities, which they clustered according to output mix, environmental conditions and level of powers, although they followed a cross-sectional approach. Choi and Park (2019) also apply this method to classify Low and Middle income countries and assess the efficiency of governmental capacity to enhance social progress. Other authors like Villalobos-Cid et al. (2016) also use cluster analysis to obtain efficiency values based on the heterogeneous performances of Chilean hospitals when the diagnosis-related groups (DRG) weights are not available. Their approach is also based on cross-sectional data however, and they do not account for a common frontier to assess the technological gaps in the healthcare system.

## Methodology

In this paper, we propose a new empirical approach based on the analysis of clusters of units relative to a common frontier, similar to metafrontier analysis (Battese et al., 2004; O’Donnell et al., 2008) and we combine it with panel data DEA (Surroca et al., 2016; Pérez-López et al., 2018). The problem that arises when we try to apply the metafrontier analysis is that it is a cross-sectional approach, so for all time periods there will be a time-specific frontier and time-specific efficiency coefficients; therefore, each time period is analyzed without any connection with the levels of activity of adjacent time periods.

To overcome this problem, we use panel data DEA proposed by Surroca et al. (2016) and Pérez-López et al. (2018). The advantage of this method over other methods proposed in the literature (like the Malmquist index) is that it enables us to estimate a single time-invariant coefficient of efficiency for the period of analysis, considering the inherent panel data structure. Also, the methodology proposed by Pérez-López et al. (2018) allows us to break down these time-invariant efficiencies into time-variant efficiency scores, obtained on a year-by-year basis. In consequence, we will not just be able to find a long-term average efficiency for the time period studied, but we can also calculate efficiency values for each year under evaluation. We extend the approach by accounting for technological asymmetries of the DMUs. An additional advantage over other parametric methods is that we do not need to assume a production function a priori, which in our case would be difficult to defend given the multidimensional nature of Ecuadorian public hospitals, plus the use of DEA methods allow us to introduce multiple inputs and outputs to estimate technical efficiencies. As far as we are aware, this methodology has not previously been applied and represents a significant innovation in the current literature.

In this study we define a non-parametric technology set. We start by obtaining homogeneous clusters of hospitals using multivariate clustering techniques. Once the groups are estimated, the common frontiers and the group frontiers can be calculated using DEA (Charnes et al., 1978; Banker et al., 1984).

As a non-parametric frontier estimation method, DEA has significant limitations that have been highlighted in the literature; the curse of dimensionality, their lack of statistical properties, and the potential impact of outliers are among the most relevant (Simar & Wilson , 2008; Cooper et al., 2006). In this respect, Pérez-López et al. (2018) state that one of the outstanding advantages of the panel data DEA is the robustness of the results to the presence of outliers and temporal random shocks; this provides a specific efficiency score, representative of the complete time period under analysis. Hence, the interpretation of the results is not far from what can be obtained from a fixed-effects parametric regression.

### Estimation of (time-invariant) panel data efficiency values for public hospitals

In our study (as in most of the applied literature quoted and presented in Table 1), we apply an input-oriented efficiency measurement. The criteria behind responds to the short-run approach of our analysis: the demand variations coming from the healthcare reforms implemented required a prompt reaction from the public hospitals, who, in the short-term have more control over their inputs to provide medical attention. Our approach also goes in line with the new healthcare strategy implemented by the MSP, directed towards equitable access and quality of treatment (Ministerio de Salud Pública, 2012). Hence, with a more efficient use of inputs, hospitals save more resources, allowing public authorities to cover a wider range of the population with the same health budget. Also, we assume a VRS model as we are dealing with heterogeneous observations.^3^ The efficiency frontier is developed by optimizing the weighted input/output ratio of each DMU, subject to the condition that this ratio can be equal to, but never exceed one for any other DMU in the data set (Charnes et al., 1978).

Let us introduce some notation. Assume that we have *I* DMUs (hospitals) $(i=1,\ 2,\ \dots ,\ I)$ classifiable in *S* clusters $\left( s=1,\ 2,\ \dots ,\ S\right)$; here are *M* outputs $[y^i_1,\ \dots ,y^i_m,\ \dots ,y^i_M \in {\mathfrak {R}}^+_M]$ produced by N inputs $[x^i_1,\ \dots ,x^i_n,\ \dots ,x^i_N\in {\mathfrak {R}}^+_N]$ in the common frontier; and $[y^{i,s}_1,\ \dots ,y^{i,s}_m,\ \dots ,y^{i,s}_M\in {\mathfrak {R}}^+_M]$ and $[x^{i,s}_1,\ \dots ,x^{i,s}_n,\ \dots ,x^{i,s}_N\in {\mathfrak {R}}^+_N]$ outputs and inputs for the *s* local frontier respectively. We denote $[y^o_1,\ \dots ,y^o_m,\ \dots ,y^o_M\in {\mathfrak {R}}^+_M]$ and $[x^o_1,\ \dots ,x^o_n,\ \dots ,x^o_N\in {\mathfrak {R}}^+_N]$ as the observed units under analysis, and likewise for the observed units in the local frontiers. We define a time variable $t\ (t=1,2,\ \dots ,\ T)$, so in the common frontier we have $[y^i_{1,t},\ \dots ,y^i_{m,t},\ \dots ,y^i_{M,T}\in {\mathfrak {R}}^+_M]$ outputs and $[x^i_{1,t},\ \dots ,x^i_{n,t},\ \dots ,x^i_{N,T}\in {\mathfrak {R}}^+_N]$ inputs; and likewise in the local frontiers. We define the following mathematical program using *contemporaneous technology * (Tulkens , 1986; Pérez-López et al., 2018), which estimates the VRS DEA (common frontier) efficiency values:

1 $$\begin{aligned}{} & {} {\mathop {\textrm{max}}_{{u^c_{0,,t},u}^c_{m,t}{,v}^c_{n,t}} {\alpha }^c_t=u^c_{o,t}+\sum ^M_{m=1}{u^c_{m,t}y^o_{m,t}}\ } \nonumber \\{} & {} \quad s.t.\ \sum ^N_{n=1}{v^c_{n,t}x^o_{n,t}}=1 \nonumber \\{} & {} \quad u^c_{o,t}+\sum ^M_{m=1}{u^c_{m,t}y^i_{m,t}-\sum ^N_{n=1}{v^c_{n,t}x^i_{n,t}\le 0;\ \ \ i=1,2,\ \dots ,\ I}}\nonumber \\{} & {} \quad u^c_{m,t}\ge 0;\ v^c_{n,t}\ge 0;\ \ m=1,2,\ \dots ,M;\ \ \ n=1,2,\dots ,N \end{aligned}$$

Where, $u^c_{m,t}$ and $v^c_{n,t}$ are weights for the outputs and inputs, for the period *t*, corresponding to the unit under evaluation; and $u^c_{o,t}$ is a scalar that can take positive or negative values, depending on the prevailing returns to scale.^4^ The problem arises when for every observed unit we obtain a time-specific frontier and a time-specific efficiency coefficient, so for every *ith *DMU we are obtaining *T* contemporaneous efficiency scores $\left( {\alpha }^c_1,\dots ,{\alpha }^c_t,\dots ,{\alpha }^c_T\right) :$ *TxM* output weights and *TxN* input weights. This implies that each time period is analyzed without any connection with the levels of activity of adjacent time periods. Also, we are conducting just an efficiency measurement for the common frontier, meaning that we are considering all hospitals in the analysis without allowing for their heterogeneity.

To overcome these issues, Surroca et al. (2016) and Pérez-López et al. (2018) propose a time-invariant panel data DEA evaluation. This technique incorporates an intertemporal frontier, which assumes a single production function for all time periods, comprising all the observations during the period of analysis. Also, it establishes a common set of weights for the complete time period. We extend this application with the incorporation of the *S* clusters generated to account for technological asymmetries. The input-oriented VRS (time-invariant) program for panel data DEA can be extended in the following way:

2 $$\begin{aligned}{} & {} {\mathop {\textrm{max}}_{u^{ti,s}_0{{,u}^{ti,s}_m,v}^{ti,s}_n} {\widetilde{\propto }}^{ti,s}=u^{ti,s}_o+\sum ^M_{m=1}{u^{ti,s}_m{\tilde{y}}^{o,s}_m}\ }\nonumber \\{} & {} \quad s.t.\ \sum ^N_{n=1}{v^{ti,s}_n{\tilde{x}}^{o,s}_n}=1 \nonumber \\{} & {} \quad u^{ti,s}_o+\sum ^M_{m=1}{u^{ti,s}_my^{i,s}_{m,t}-\sum ^N_{n=1}{v^{ti,s}_nx^{i,s}_{n,t}\le 0;\ \ \ i=1,2,\ \dots ,\ I};\ \ \ \ s=1,2,\dots ,S}\nonumber \\{} & {} \quad u^{ti,s}_m\ge 0;\ v^{ti,s}_n\ge 0;\ \ m=1,2,\ \dots ,M;\ \ \ n=1,2,\dots ,N \end{aligned}$$

Note that ${\widetilde{\propto }}^{ti,s}$ is an average value that represents the one time-invariant efficiency coefficient for hospital under observation ‘o’ while comparing it with its respective cluster *s*; ${\tilde{y}}^{o,s}_m=\sum ^T_{t=1}{y^{o,s}_{m,t}/T}$ is the average value, corresponding to output *m* in hospital ‘o’ forming part of cluster *s*, for the complete time period *T*; and ${\tilde{x}}^{o,s}_n=\sum ^T_{t=1}{x^{o,s}_{n,t}/T}$ is the average value, corresponding to input *n* in hospital ‘o’ forming part of cluster *s*, for the complete time period *T*. By applying the programs for the *S* clusters, we obtain *MxI* output weights and *NxI* input weights corresponding to the *I* hospitals classified in the *S* clusters. According to Pérez-López et al. (2018), besides obtaining a time-invariant common set of weights for each hospital, program (2) has three additional properties: (1) it is less dependent on the specific values of the variables in one particular year; (2) it ensures that no changes in the valuation system (input and output weights) take place across time periods; and (3) the consideration of average values does not imply any loss of information.

To compare the time-invariant cluster efficiencies, relative to the time-invariant common frontier efficiencies, we need to define the technology reference for the entire sample of units. This way, we obtain the following program:

3 $$\begin{aligned}{} & {} {\mathop {\textrm{max}}_{u^{ti}_0{{,u}^{ti}_m,v}^{ti}_n} {\widetilde{\propto }}^{ti}=u^{ti}_o+\sum ^M_{m=1}{u^{ti}_m{\tilde{y}}^o_m}\ }\nonumber \\{} & {} \quad s.t. \sum ^N_{n=1}{v^{ti}_n{\tilde{x}}^o_n}=1 \nonumber \\{} & {} \quad u^{ti}_o+\sum ^M_{m=1}{u^{ti}_my^i_{m,t}-\sum ^N_{n=1}{v^{ti}_nx^i_{n,t}\le 0;\ \ \ i=1,2,\ \dots ,\ I}}\nonumber \\{} & {} \quad u^{ti}_m\ge 0;\ v^{ti}_n\ge 0;\ \ m=1,2,\ \dots ,M;\ \ \ n=1,2,\dots ,N \end{aligned}$$

Now we have ${\widetilde{\propto }}^{ti}$, which is an average value that represents the time-invariant efficiency coefficient for the hospital under observation; ${\tilde{y}}^o_m=\sum ^T_{t=1}{y^o_{m,t}/T}$ is the average value, referring to unit ‘o’ under observation, corresponding to output *m*, for the complete time period *T*; and ${\tilde{x}}^o_n=\sum ^T_{t=1}{x^o_{n,t}/T}$ is the average value, corresponding to input *n* for unit ‘o’, for the complete time period. For these efficiencies we are assessing the average level of efficiency of the complete time period with no isolated consideration of any specific time period in relation to the local and common frontier.

Finally, the time-invariant TGR comes straightforwardly as:

4 $$\begin{aligned} TGR=\frac{{\widetilde{\propto }}^{ti}}{{\widetilde{\propto }}^{ti,s}} \end{aligned}$$

The minimized value of ${\widetilde{\propto }}^{ti,s}$ that solves the cluster *s* linear program is no greater than the minimized value of ${\widetilde{\propto }}^{ti}$ that solves the metafrontier linear program, hence, the metafrontier will never lie below any of the group frontiers. This way, the TGR measures how close a group frontier is to the metafrontier, representing the restrictive nature of the production technology. The closer it gets to 1, the higher the efficiency in operations that can be achieved (Mitropoulos et al., 2015).

###  Estimation of (time-variant) panel data efficiency values for public hospitals

In their paper, Pérez-López et al. (2018) demonstrate that it is possible to derive time-variant efficiency scores from the previous time-invariant ones in order to obtain the variations in efficiency coefficients during the different time periods, maintaining the robustness of the values over time. If we consider one input example, under an input-oriented approach they demonstrate that:

$$\begin{aligned}{\widetilde{\propto }}^{ti}={\widetilde{\alpha }}^{tv}_1\frac{x^o_{n,1}}{\sum ^T_{t=1}{x^o_{n,t}}}+\dots +{\widetilde{\alpha }}^{tv}_t\frac{x^o_{n,t}}{\sum ^T_{t=1}{x^o_{n,t}}}+\dots +{\widetilde{\alpha }}^{tv}_T\frac{x^o_{n,T}}{\sum ^T_{t=1}{x^o_{n,t}}}\end{aligned}$$

5 $$\begin{aligned} {\widetilde{\propto }}^{ti}\mathrm {=\ }\sum ^T_{t=1}{{\widetilde{\alpha }}^{tv}_tw_t\mathrm {\ }} \end{aligned}$$

So that time-invariant panel data efficiencies are equal to the weighted average of the time-variant panel data efficiency coefficients. We can extend this same application to obtain time-variant panel data efficiency coefficients for every *s* cluster acquired. The mathematical representation is straightforward:

$$\begin{aligned}{\widetilde{\propto }}^{ti,s}={\widetilde{\alpha }}^{tv,s}_1\frac{x^{o,s}_{n,1}}{\sum ^T_{t=1}{x^{o,s}_{n,t}}}+\dots +{\widetilde{\alpha }}^{tv,s}_t\frac{x^{o,s}_{n,t}}{\sum ^T_{t=1}{x^{o,s}_{n,t}}}+\dots +{\widetilde{\alpha }}^{tv,s}_T\frac{x^{o,s}_{n,T}}{\sum ^T_{t=1}{x^{o,s}_{n,t}}}\end{aligned}$$

6 $$\begin{aligned} {\widetilde{\propto }}^{ti,s}\mathrm {=\ }\sum ^T_{t=1}{{\widetilde{\alpha }}^{tv,s}_tw^s_t\mathrm {\ }} \end{aligned}$$

### Hypotheses

To answer the research question posed in this paper, we need to look for a way to determine whether the healthcare efficiency performance of the public hospitals in Ecuador has undergone a significant change, which might be partly driven by the health reforms introduced under the new Correa government. In order to do so, we apply a before-after approach and divide the time period under study into two sub-periods. We estimate the time-variant and time-invariant efficiencies by applying the linear programs (2) and (3) to each sub-period. We consider 2008 as a potential turning point, when the new constitution was introduced and marked the beginning of several health reforms.

Thus, we define ${\alpha }^{tv}_{p1}$ as the time-variant efficiencies for the period 2006–2008, and ${\alpha }^{tv}_{p2}$ as the time-variant efficiencies for the period 2009–2014.^5^ If the reforms that came after the new constitution affected the amount of inputs consumed in the health production process, for example, and if the new amount of patients attended caused an over-demand for healthcare services increasing the resources needed to treat them, then this would probably be reflected in a change in the average public hospital efficiency. Thus, if the health reforms negatively affected the efficient performance of public hospitals, then we should see a significant decrease in their average efficiency ($\widetilde{\alpha }$), so ${\widetilde{\alpha }}^{tv}_{p1}\mathrm {\ }>\ {\widetilde{\alpha }}^{tv}_{p2}$. We will test this hypothesis by means of two statistical tests. The first one is the Wilcoxon signed rank test for dependent samples, which is a non-parametric test that does not need the assumption of normal distributions and has often been used in the literature to test significant differences in ordinal variables (O’Neill et al., 2008; Prior and Surroca, 2010). For the second test, we consider a method that provides us with more accurate information, namely the Li (1996) test for unknown distributions.

The Li (1996) method relies on kernel smoothing to non-parametrically estimate the density functions corresponding to ${\alpha }^{tv}_{p1}$ and ${\alpha }^{tv}_{p2}$ indices. However, Simar and Zelenyuk (2006) argue that in order to test the efficiency values estimated, the Li (1996) method has to be modified in several ways (see Simar and Zelenyuk 2006).They provide consistent bootstrap estimates of the $\rho$ values of the Li (1996) test and encourage its empirical application in efficiency measurement research. The estimation of the time-variant efficiency values will help us to back up these hypotheses and find the trends in healthcare efficiency over the years.

Finally, in order to support these results and provide statistical evidence of the effect of the 2008 reform on the time-variant efficiency, we apply three different multivariate regressions. First, to obtain robust estimates that permit a valid inference, we follow Simar and Wilson (2007) truncated regression (Algorithm # 1). To capture the effect of the introduced health reform, we use a time dummy that takes the value of 1 for the years 2009–2014, and 0 otherwise. We also control for the technological heterogeneity with two dummies for low and intermediate-tech clusters, respectively. At last, we control for hospital and municipal variables that can potentially affect hospital efficiency.^6^As robustness checks, we run a censored (tobit) model and an OLS estimation (Banker and Natarajan, 2008), and compare the estimated marginal effects.

## Data

For the purpose of the study, we use the Annual Survey of Hospital Beds and Discharges and the Survey of Health Activities and Resources provided by INEC for the years 2006–2014. We consider the information on public hospitals excluding from the sample psychiatric, dermatology and geriatric hospitals.^7^

In a first stage, we use factor analysis with various correlated input variables available for all time periods in our dataset that can best approximate the health resources which contribute to the production of health. Based on the interdependencies of these variables we obtain a reduced set of uncorrelated variables called factors. With these new factors we run a hierarchical cluster analysis, which is a multivariate technique that seeks to cluster a set of *I* units into *S* groups depending on the similarities between them, so that (1) each unit is in one and only one of the groups; (2) every unit is classified, and (3) each group is internally homogeneous. The advantage of running a factor analysis previous to the clustering technique is that we can eliminate the dimensions which we are (practically) sure are only noise. Therefore, we retain the components responsible for a very high percentage of the inertia; thus, the hierarchy obtained is considered to be more stable and clearer (Husson et al., 2010).

In order to define the number of clusters to be constructed, we need to consider both the measure of similarity and the clustering method. We use the Euclidean distance as it is the most commonly used method in the literature to measure similarity, and the Ward hierarchical clustering method, which has the advantage of maximizing intra-group homogeneity and inter-group heterogeneity. Additionally, it is robust to outliers and groups are not too dissimilar in size (Balaguer-Coll et al., 2013). Finally, the Caliñski and Harabasz (1974) stopping rule was used in order to determine the number of clusters.

With this approach, we improve the standard applications used so far in the healthcare efficiency literature.^8^ Thus, we use multivariate statistical analysis to generate specific clusters, differentiated by their technological endowment rather than size, and by applying factor analysis in the first stage, we make the variables independent of each other, avoiding potential correlation problems in the following analyses. The variables used to obtain the technology clusters are described and summarized in the Online Appendix.

Given that factor and clustering analyses are cross-sectional techniques, we face the problem of an inconsistent grouping of DMUs for each year, which makes the efficiency values challenging to obtain for each group over the years. To overcome this problem, we take the average values of each variable over time and perform the multivariate analyses (factor and cluster analyses). This yields ${\tilde{S}}_1, {\tilde{S}}_2,\dots, {\tilde{S}}_c$ groups shaped by the average technological endowment of each hospital. Despite some limitations that it could bring to the analysis, our goal is to obtain average efficiency estimations with the programs (2) and (3), making this approach the best fit to our empirical application.

The second stage of the analysis measures the average efficiency of hospitals over the years using programs (2) and (3) in both the group frontier and metafrontier, but before turning to the results, we must define the inputs and outputs to be used.

### Inputs

There is common agreement on the use of inputs in the literature (O’Neill et al., 2008). To avoid potential problems of dimensionality, we aggregated the different health resources (described in the Online Appendix) into four input variables: the total number of beds (totcam), hospital equipment and infrastructure (variables eq2 to eq8), the number of physicians (variables m1 to m7) and the number of professional healthcare personnel other than physicians that work in the hospital (proftit to p2).

The number of hospital beds has been widely used in the literature as a proxy for hospital size and capital investment (O’Neill et al., 2008); we also include variables that describe equipment and infrastructure of hospitals to account for this.^9^ The majority of studies include the number of clinical staff as a proxy for labor costs (O’Neill et al., 2008; Cantor and Poh, 2018).^10^

### Outputs

Most published research uses some variant of intermediate outputs in terms of patients treated or number of inpatient days hospitalized (Hollingsworth, 2008). To measure the final production of the health of public hospitals we use the number of discharges as an output variable. However, we need a method to adjust outputs for patient heterogeneity (i.e. case mix) as not all conditions can be treated with the same amount of resources and not all hospitals have the means nor the capacity to treat serious illnesses; therefore, if not taken into account, hospitals with a more complex case mix are likely to receive lower efficiency scores. By including a case-mix weight, we are explicitly designing groups that provide comparable resource intensity care, and we can also distinguish the hospitals treating more severely ill patients, requiring more inputs from hospitals treating less resource-intensive patients (Valdmanis et al., 2017). Perhaps the most successful approach is the use of DRG classification which categorizes patients according to diagnosis, treatment and length of stay. However, in developing countries this tool is not fully (or even partially) implemented, which limits the efficiency of the evaluation (Villalobos-Cid et al., 2016). This constraint holds true for the Ecuadorian case, which leads us to apply alternative approaches based on the available data, and which have been previously applied in the literature.

Therefore, to treat the severity of cases in this study, we use the three-digit *International Statistical Classification of Diseases and Related Health Problems* (ICD-10) to construct the case-mix weight, following the approach developed by Herr (2008). This approach relies on the assumption of a correlation between the length of stay and the severity of illness, so the idea is that the more days of patient stay, the more severe the disease and the more resources are used.^11^ Other authors such as Herr et al. (2011), Herwartz and Strumann (2012, 2014) and, Varabyova and Schreyögg (2013) suggest using this approach in the absence of the DRG classification.

For the sake of simplicity, the descriptive statistics for the years 2006 and 2014 for each cluster are presented in Table 2.^12^ We ran two outlier detection methods on the data. The first was proposed by Prior and Surroca (2010), and the second one is based on Andrews and Pregibon (1978) and Wilson (1993).^13^

**Table 2:** Inputs and Outputs, descriptive statistics$^a$

	2006						2014					
	Cluster 1 (High)		Cluster 2 (Intermed.)		Cluster 3 (Low)		Cluster 1 (High)		Cluster 2 (Intermed.)		Cluster 3 (Low)	
	N=8		N=14		N=143		N=6		N=15		N=148	
	Median	SD	Median	SD	Median	SD	Median	SD	Median	SD	Median	SD
*Output*												
Weighted Discharges	4505	2623.6	782	3053.9	814	2076.5	6344	5372.8	977	3943	984	2547.1
*Inputs*												
Physicians	134	76.2	17	82.7	16	34	275	198.5	27	196.4	29	80.3
Beds	263	118.8	33	165.8	21	101.6	277	232.9	32	213.5	29	100.4
hospital personnel	319	210	22	188.9	23	108.7	661	344.4	51	365.5	51	169.5
Equipment and Infrastructure	152	56.5	40	65.6	32	57.8	358	188.2	52	69.9	48	104.4

$^a$MANOVA tests indicated that the differences between groups are statistically significant. The observations for the clusters over the years differ slightly due to some missing information in the dataset. However, the methodology applied is robust to these discrepancies

Comparing the levels of input mix across the clusters presented in Table 2, we can observe that the first cluster accounts for high levels of technological endowment. As expected, the hospitals belonging to this cluster attend to a much broader share of patients in the country, even though their number is remarkably lower than the other clusters. The second cluster is shaped by hospitals with an intermediate level of technology, and on average, is not very far from the final cluster. Finally, the last cluster comprises hospitals with a low level of technological endowment. Hence, we define our clusters based on the average amount (or the level) of hospital resources (technological endowment) of each group. Being the high-tech cluster the one that presents (on average) the highest technological endowment. Conversely, the intermediate and low-tech clusters are those that present (on average) a medium and lowest technological endowments, respectively. It is important to note the marked difference in the number of hospitals in the high-tech cluster, relative to those in the high and intermediate technology clusters; this difference highlights the profound technological heterogeneity, not just in terms of the large asymmetries present in the public healthcare system, but also in its notable share of technologically lagging hospitals.

## Results and discussion

Table 3 shows the time-invariant efficiencies resulting from programs (2) and (3), both for the metafrontier and for the respective local frontiers. Looking at the metafrontier average efficiency, we can see that overall the hospitals present very low efficiency scores. The value of 0.3894 would mean that to be fully efficient Ecuadorian hospitals have to reduce their input consumption by 61.06%, representing more than half of resource consumption. Additionally, some hospitals present a minimum level of inefficiency as low as 0.1088, which represents a severe problem of inefficiency in the system. However, we cannot draw hasty conclusions in this manner, as other hospitals present high levels of efficiency, showing profound asymmetries inherent in the system.

**Table 3:** Time-invariant efficiencies, summary statistics

	Mean	Median	SD	Min	Max	TGR	N
Metafrontier	0.3894	0.3838	0.1483	0.1088	0.9318		1674
Cluster 1 (High)	0.6479	0.6371	0.1926	0.4135	0.9095	0.5433	81
Cluster 2 (Intermediate)	0.5623	0.5781	0.2434	0.2269	0.9339	0.7183	189
Cluster 3 (Low)	0.4269	0.4282	0.1502	0.113	1	0.9176	1404

When we look at the efficiencies obtained for each cluster, the results are quite different. Overall, the average (group) efficiencies in cluster 1 and cluster 2 are much closer to the frontier than those of cluster 3. Hence, when considering the technological differences between hospitals, on average, high-technology ones are making better use of inputs than low-technology ones. The differences compared with a single frontier estimation are remarkable, highlighting the importance of accounting for the heterogeneity of the system. Assessing public healthcare systems within a homogeneous framework of comparable hospitals is therefore questioned, especially Ecuadorian public hospitals whose differences have been worsened by its historical economic and political situation.

Regarding the TGR, results seem to be counterintuitive. Cluster 1 and cluster 2 present the widest gap between group efficiencies and the metafrontier, suggesting that they are more constrained by the nature of their production environment, and efficiencies in these clusters are further away from the metafrontier than those of cluster 3, whose efficiencies are very similar to the metafrontier. It would appear that the output complexity is not properly captured by our data, which is leading the metafrontier to fail in the measurement of the unconstrained production defined by Battese et al. (2004).

**Fig. 4:**
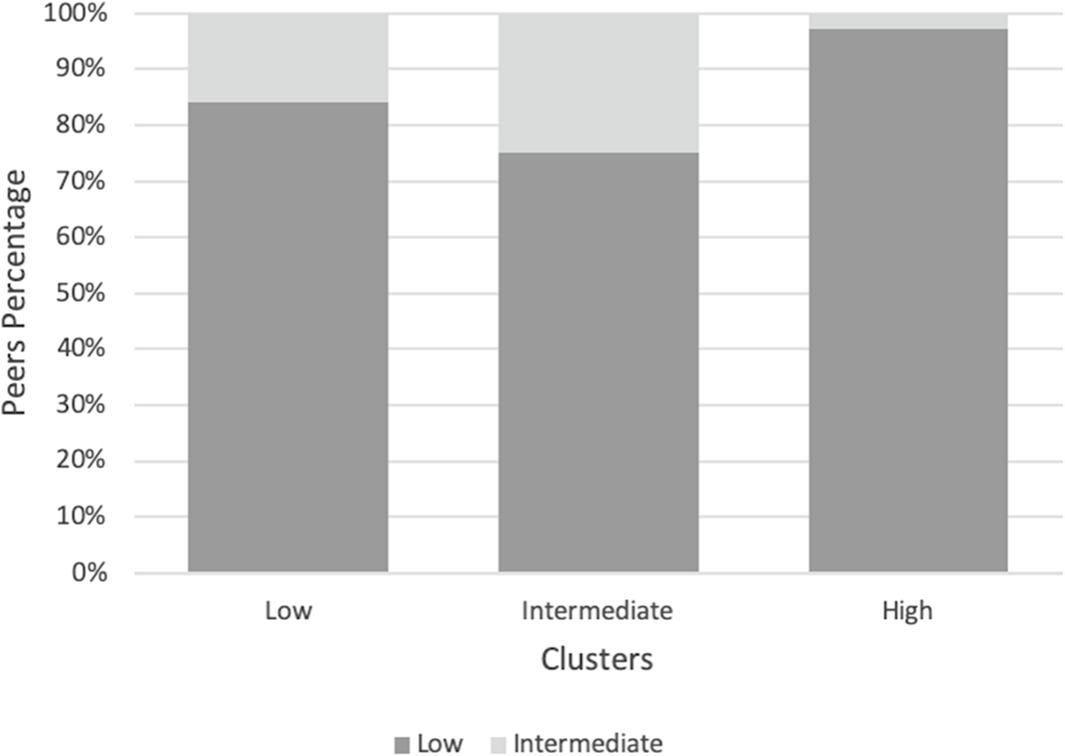
Percentage of peer participation relative to the metafrontier

To investigate this question, we plotted the hospitals’ peer participation for each cluster with respect to the metafrontier in Fig. 4.^14^ The criteria goes as follow: for every inefficient DMU belonging to each cluster (x-axis) there is another efficient DMU in the metafrontier that serves as peer to be compared with, the total percentage of these peers for every cluster is plotted in the y-axis. Thus, a hospital should be compared with a peer that has at least the same technological endowment. The figure shows that the hospitals belonging to each group take as reference hospitals for production (in the metafrontier) those that belong to the low and intermediate technology hospitals. For example, 97% of the reference units (peers) for the inefficient high-tech hospitals have the lowest level of technological endowment, while the remaining 3% have an intermediate technological level. These results show that, even though we tried to capture the complexity of the cases through length of stay, we are still not able to find a proper case mix that accounts for the full complexity of the patients treated. In addition to this, the profound heterogeneity in the system is also playing a significant role in these results. This is because the few high-tech hospitals treat more than five times as many patients as the low-tech hospitals, leading to saturation of their resources, and as a result, jeopardizing their performance.

Thus, in a situation of limited data availability, and where the marked heterogeneity in the system is preventing a proper metafrontier evaluation, we need a method that considers the technological differences of the system to avoid misleading conclusions. In this context, it is reasonable to assume that the hospitals that have the higher technology can only be compared with each other, meaning that the only reference units they will be compared with are those that have the same level of technology. It would be unreasonable to think that low-tech hospitals that do not have the same resources can be a reference for those in the high-tech group. Similarly, the intermediate technology hospitals would be compared with each other and with the high-tech hospitals but cannot be compared with the low-tech hospitals. This idea leads us to construct a new metafrontier, taking into account what Banker and Morey (1996) call a shift in the production frontier. Banker and Morey (1996) state that different hospitals have different characteristics that need to be considered in the efficiency analysis; unfortunately, these characteristics cannot always be observed in practice. This is especially important when the impact of a factor (like technology endowment in our case) varies substantially across demographic, competitive or other contingent environments.

**Fig. 5:**
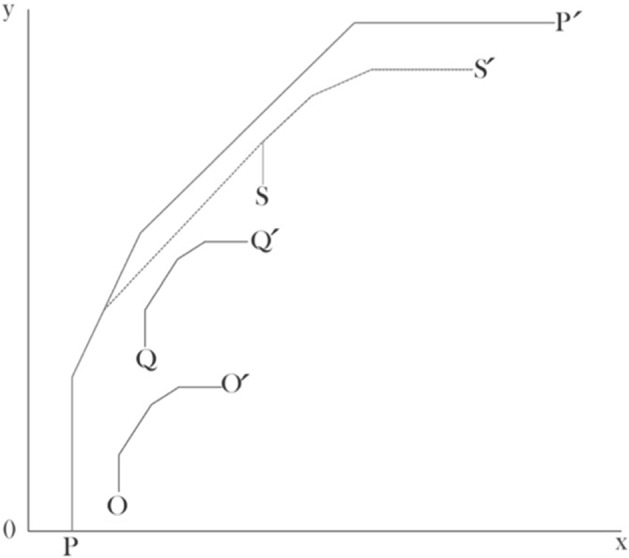
Newly constrained metafrontier

Based on the idea of Banker and Morey (1986, 1996), in this research we propose a method to construct a new metafrontier. We will assume that the hospitals studied here cannot be compared with hospitals endowed with less technology. The shift of the production frontier leads us to define a new metafrontier. The intuition underlying our approach is displayed in Fig. 5, where the metafrontier PP’ depicts the prior metafrontier where all hospitals are benchmarked against each other, meaning that we take all clusters into consideration $(s=1,2,\dots ,S)$ The shift is produced when we constrain the metafrontier to be benchmarked only against those hospitals with similar or higher technology. Here, we imply that the high-tech hospitals, represented in the frontier SS’, will only be compared with each other, so they do not present a TGR. The new metafrontier is depicted by PS’.

This idea also follows the line of selective convexity developed by Podinovski (2005). This concept states that the DMUs can be used to form convex combinations provided that they are different only in the inputs and outputs for which the convexity assumption is accepted. Additionally, this author demonstrates that under the free disposability assumption, selective convexity generalizes the inequalities stated by Banker and Morey (1986).

Using this approach, we solve the new metafrontier applying the linear program (3) under three scenarios where the three hospital groups analyzed are benchmarked according to their level of technology: $s=1;s=1,\ 2$; and $s=1,2,3$. The sum of these three estimations together result in the new metafrontier depicted by PS’.

The results obtained with the new metafrontier are presented in Table 4. This new estimation of a metafrontier allows us to determine the technological gap of the three clusters of hospitals, considering the asymmetries in the system not just in the local frontiers, but also in the common frontier. As we can see, now cluster 3 accounts for the highest technological gap relative to cluster 2, which shows a shorter distance from the metafrontier. The difference in TGR for the low-tech hospitals shows that, given their limited levels of technology, they can achieve a maximum efficiency of 90% of what is feasible with the highest level of technology available. We have to be aware that overall, the level of efficiency in the system is rather low, which can be explaining the short distances in the TGR.

**Table 4:** Time-invariant efficiencies, summary statistics (new constrained metafrontier)

	Mean	Median	SD	Min	Max	TGR	N
Metafrontier	0.4215	0.3962	0.1731	0.113	0.9339		1674
Cluster 1 (High)	0.6479	0.6371	0.1926	0.4135	0.9095	1	81
Cluster 2 (Intermediate)	0.5623	0.5781	0.2434	0.2269	0.9339	0.9994	189
Cluster 3 (Low)	0.4269	0.4282	0.1502	0.113	1	0.9176	1404

The advantage of applying the panel data DEA technique in our analysis is that it provides an additional tool with the time-variant efficiencies, allowing us to obtain the trends in efficiency of the hospitals analyzed without losing the robustness of the previous results. This enables us to shed some light on the exact year when the efficiencies started to decrease and have a clearer idea of whether this could have been a direct result of the healthcare policies implemented.

**Fig. 6:**
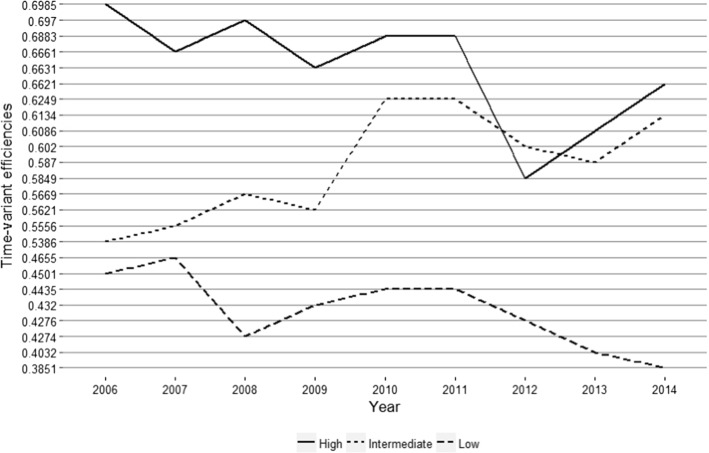
Evolution of time-variant panel data efficiencies

Following this idea, Fig. 6 shows the time-variant efficiencies obtained for the group frontiers in the period under analysis. Some interesting facts can be garnered from this figure. First, the efficiencies of the high-technology hospitals show peaks of performance in the first period of analysis, but their behavior does not seem to change immediately after 2008. This could be because historically there has always been a limited number of high-technology public hospitals in Ecuador and they attend to most of the patients in the Ecuadorian health network, which has not changed over the years.

In contrast, low and intermediate technology hospitals show an increase in 2008 and 2009. This improvement might be reflecting the positive effect on efficiency due to the optimization of spare resources and capacity, likely misused prior to the reforms. The investment deployed in the health sector could also be a potential driver of this rise in efficiency. In the short run, the increase in the health budget could have triggered higher productivity in the system; for example, physicians, managers or general health personnel may have been motivated by potential salary raises.

There seem to be two particular years when the behavior of the three clusters changes. The first one goes from 2010 to 2011, when all groups shifted from an increasing to a constant efficiency. The second one relates to the year 2011, a year in which efficiency declined severely. Two facts are worth noting in these two periods. In 2010 there was a social security reform that allowed the insured population to extend insurance to their children under the age of 18, which might have halted the increase in performance of all clusters of hospitals due to the sudden rise in patients. Moreover, in 2011 Ecuador held a referendum, which (among other matters) included the approval of a law for the deprivation of liberty for employers who do not affiliate workers within a maximum period of 30 days (Orellana et al., 2017). This new law, added to the new free services, caused an increase in demand that De Paepe et al. (2012) refer to as a “demand crisis” due to the sudden rise of the insured population, especially in larger cities. This increased demand might be a substantial cause of the pronounced decline seen in all three groups of hospitals.

It should be noted that we are not claiming that this decline in efficiency was actually caused by the increase in demand, but in this study we offer strong empirical evidence to suggest that this could be a strong driver.

### Hypotheses tests

In order to corroborate the hypothesis of a significative change in the average efficiency performance after 2008, Table 5 shows the descriptive statistics of the time-invariant efficiency values for the sub-periods $p_1\ (2006-2008)$ and $p_2\ (2009-2014)$. The average values for both the group frontiers and the metafrontiers seem to have decreased. This decline could be signaling that the government’s policies indeed had a negative effect on the efficiencies of all groups of hospitals in the public health system.

**Table 5:** Time-invariant efficiencies for each sub-period, summary statistics

		Mean	Median	SD	Min	Max	N
2006–2008	Cluster 1 (High)	0.7694	0.8942	0.219	0.4415	1	27
	Cluster 2 (Intermed.)	0.6991	0.79	0.2701	0.2353	1	63
	Cluster 3 (Low)	0.5226	0.5069	0.1878	0.1519	1	468
2009–2014	Cluster 1 (High)	0.6985	0.719	0.2178	0.3921	0.9656	54
	Cluster 2 (Intermed.)	0.5816	0.6003	0.2522	0.234	0.9664	126
	Cluster 3 (Low)	0.5144	0.5299	0.165	0.1178	1	936

**Fig. 7:**
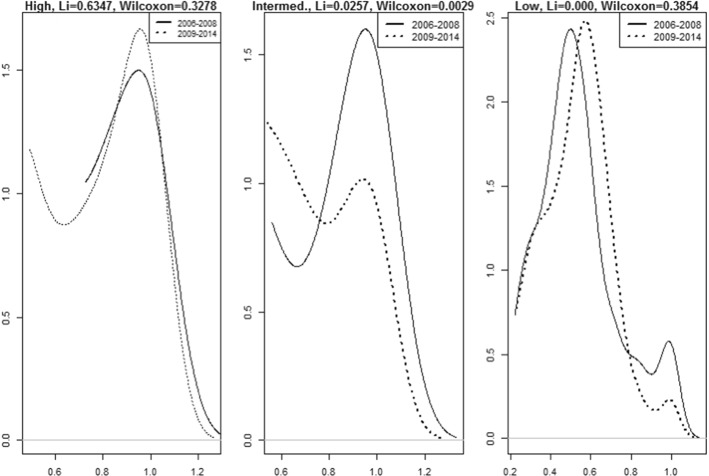
Density plots 2006–2008 vs 2009–2014. The Li and Wilcoxon scores correspond to Simar and Zelenyuk’s extension of the Li test and the Wilcoxon signed rank test *p*-values, respectively.

We provide evidence of this possibility in Fig. 7, where we plot the smoothed densities of the group efficiencies. The Li (1996) and Simar and Zelenyuk (2006) test, and the Wilcoxon signed rank test *p*-values for the efficiency scores of the group frontiers in both sub-periods are depicted along with the graph. Based on the information provided by the extended Li (1996) and Simar and Zelenyuk (2006) test–which has proved to provide more accurate and reliable results in several fields applied to efficiency measurement (Pastor and Tortosa-Ausina, 2008; Li et al., 2009; Balaguer-Coll et al., 2013)—the null hypothesis of equal distributions is rejected for all groups except the high-tech hospitals. The evidence presented here falls in line with the above-mentioned results. Apparently, the high-tech hospitals have not experienced a significant decrease in efficiency since 2008, but rather the low and intermediate technology hospitals are the most affected.

Table 6 shows the results of the marginal effects estimated using the OLS, tobit and truncated regressions. The results are significant and comparable. The empirical evidence supports the findings that after the reform of 2008 (Ref_2008) there was a negative impact on the average efficiency of public hospitals, the estimated marginal effects for the dummies introduced for the low and intermediate-tech hospitals are also negative and statistically significant, and the size of this effect is bigger (in absolute terms) for those hospitals belonging to the lowest technological group. This is evidencing that the decrease in efficiency is negatively associated with the technological endowment of public hospitals, supporting the findings of our hypotheses tests.

**Table 6:** OLS, tobit, and truncated regressions$^a$

	OLS	Tobit	Truncated
Ref_2008	$-$0.034***	$-$0.034***	$-$0.032***
	(0.009)	(0.009)	(0.008)
Low-tech dummy	$-$0.266***	$-$0.274***	$-$0.230***
	(0.022)	(0.024)	(0.019)
Intermed-tech dummy	$-$0.072**	$-$0.074**	$-$0.064***
	(0.030)	(0.036)	(0.023)
Hospital controls	Yes	Yes	Yes
Municipality controls	Yes	Yes	Yes
N	1674	1674	1629

^a^The dependent variable is the time-variant efficiency. Marginal effects and related bootstrapped standard errors in parentheses computed using 2000 draws. ***p<0.01,**p<0.05, *p<0.1

The evidence provides an initial picture of the performance of the public healthcare system in Ecuador. Despite the (overall) low levels of efficiency, there seem to be some potential factors that are causing the decline in performance, and it appears to be affecting mainly the less technological hospitals. The literature on healthcare efficiency measurement offers some explanations in this matter. For example, the non-significant change in efficiency of high-tech hospitals might reflect their capacity to treat complex cases in a more efficient manner; the concentration of specialized physicians and equipment in these hospitals might be allowing them to cope better with the increasing volume of patients than low and intermediate technology hospitals would be able to, suggesting the existence of a process of learning-by-doing in high-tech hospitals (Gobillon and Milcent, 2013).

Cream skimming could also be playing an important role in this difference. The referrals to private institutions might not be alleviating the consumption of inputs in public hospitals, given that complex cases remain in the public sector and tend to stay for a longer time (Cheng et al., 2015) and demand more health services than necessary if they are covered by public insurance (Orellana et al., 2017). Given that high-tech hospitals could be showing a process of learning-by-doing, the low-tech hospitals could be more affected.

These results suggest some recommendations for public authorities and policymakers. The drop in efficiency coincides with two of the most far-reaching reforms in social security and promotion of universal coverage. The authorities should keep in mind that public hospitals need to have the necessary means in the short term with which to adapt to a sudden increase in the insured population, and that the effect on their efficiency can depend on the type of hospital where the resources are allocated. Our findings provide an initial motivation to look deeper into this matter and formulate focused policies that encourage better allocation of resources to hospitals that might be suffering most from these negative effects. The study also reveals a positive effect on efficiency in 2008 and 2009. Academics and authorities should further explore this effect and identify the sources of this improvement, which can also bring strong policy recommendations to enhance the healthcare system. Although the potential causes of efficiency variation do not fall within the scope of this study, we offer readers a wide range of unexplored research ideas in this field, and strongly encourage further investigation.

## Conclusions

The present study aimed to analyze whether the public health reforms introduced in Ecuador since 2008 have had a significant effect on the efficiency of its hospitals. To take into account the technological differences of Ecuadorian hospitals, we use a two-stage analysis, wherein the first stage we apply a multivariate factor analysis and clustering techniques to obtain homogeneous groups characterized by their technological endowment. In the second stage, we propose a combined metafrontier panel data DEA method that yields robust efficiency scores, representative of the complete time period.

The results show considerable inefficiency in the whole period when we contemplate all hospitals in a common frontier. However, when they are disentangled into technologically different groups, the difference is remarkable. Compared to their respective local frontiers, high and intermediate technology hospitals seem to be performing rather better than low-tech hospitals, which present an average efficiency very similar to the metafrontier. These results highlight the importance of considering the heterogeneities inherent in the system; if not taken into account, these heterogeneities can bias the results and lead to misleading conclusions.

The TGR for the respective groups seems to be counterintuitive as low-technology hospitals show a shorter distance from the metafrontier. However, conventional methods in the literature applied to developed economies cannot be simply translated to developing countries, which have different economic and social structures. The lack of good quality data in developing countries such as Ecuador, and the deep heterogeneity of their systems, complicates the application of conventional models such as the metafrontier production. In this study, we propose an approach to re-define the metafrontier function by means of concepts such as frontier shift and selective convexity, introduced by Banker and Morey (1986, 1996) and Podinovski (2005).

Our approach assumes that in such a technologically heterogeneous context, we cannot compare groups of hospitals, as those with lower technology will not be able to perform at the same level as those with much higher technology. Hence, high-tech hospitals can simply be benchmarked against each other. This way, we find that given these constraints, the lowest technology hospitals can perform with a maximum efficiency of 90% of what they would be able if they had the maximum technology available.

The empirical results of the efficiencies run before and after the new constitution in 2008 show an evident decline in the average efficiency of the public hospitals. Moreover, we find that 2008 had no significant effect on the trend of high-technology hospitals, whereas a statistically significant decrease in efficiency is found in low and intermediate technology hospitals. A short-run effect, shown as an increase in efficiency, is also observed among low and intermediate technology hospitals. This improvement may be due to the public investment made in Ecuador since the beginning of Rafael Correa’s mandate, which might have had an immediate effect in the system. With the immediate increase in their budget, hospital managers or medical personnel could have been motivated to increase their productivity. Additionally, the slight increase in demand prior to the most far-reaching health reforms in social insurance could have allowed some hospitals to make better use of spare capacity and medical resources, which may have been inefficiently utilized. Nonetheless, this effect was interrupted in 2010 and further reversed in 2012, coinciding with the Ecuadorian health reform that guaranteed social insurance for all workers in a dependency relationship with their employer. The evidence suggests that the sudden influx of patients generated by this reform could have had a direct effect on the observed drop in efficiency. These hypotheses are not firm conclusions, but they open up new research questions and encourage future inquiry in this field.

This study can be considered as a first step to further research to more deeply explore the potential determinants of the efficiency behavior in Ecuador’s healthcare system. This strand of research can be of significant relevance to implement focused healthcare policy better able to allocate resources in the system and alleviate the saturation that might be occurring in a limited number of hospitals that receive more than half the demand for medical services in the country.

The methodology implemented can also serve as a reference to apply to other heterogeneous realities such as that of Ecuador, where good quality data may not be available to implement classical efficiency measurement approaches. In this regard, further work can be conducted in Latin American countries. The literature has found that high territorial heterogeneity in developing countries, particularly in Latin America, shapes economic and social inequalities that have characterized the region over the years (Cuadrado-Roura and Aroca, 2013) and many of them lack good healthcare data (Villalobos-Cid et al., 2016).

Further methodological innovations can also be implemented. For example, we can consider a similar approximation by adapting a stochastic frontier analysis (SFA) to our dataset. However, the main setback of SFA approaches is that they rely on a production function that has to be defined a priori (O’Neill et al., 2008) and that cannot be simply proposed in the context of a developing country. Technical efficiency measures are very sensitive to the choice of functional specification (Giannakas et al., 2003), which can be misleading if not correctly specified. Future work should focus on defining the theoretical framework of a proper production function to provide the background for empirical applications.

Finally, some limitations of this work should be noted. First, the limited quality and availability of data has constrained the sample to the years addressed here and necessitated alternative data treatment approaches. The need to take into account a wider time period is highlighted, which would provide useful information on how the country has been adapting to these relatively new reforms over the years.

## Supplementary Information

Below is the link to the electronic supplementary material.

Supplementary file 1 (pdf 97 KB)

## Footnotes

1 Ecuadorian Social Security Institute (IESS), Social Security Institute of the Armed Forces (ISSFA) and Social Security Institute of the National Police (ISSPOL).

2 Any unit of analysis can be labeled as a DMU, for example individuals, departments, firms, municipalities, or, in the case of this study, hospitals.

3 This is also tested in the empirical application with the Simar and Wilson (2011) returns-to-scale test.

4 The output and input weights *u* and *v* can be obtained by solving the “primal” (or multiplier) form of the DEA program. They provide extra information in that they can be interpreted as normalized shadow prices (Coelli et al., 2005).

5 We apply the same procedure to the time-invariant efficiencies of each cluster obtained.

6 Hospital controls comprehend the logged occupancy rate, mortality rate, and market share; whilst, as municipal controls, we use the logged gross value added, density, municipal mortality and the share of population over 65 years old.

7 Given some irregularities in the information for some hospitals and missing data for some years, we retrieve a non-balanced panel data.

8 The literature has mostly used hospital size as a simple grouping criteria, proxied by variables like total beds or patients attended ( see for example,Arocena and García-Prado 2007, Mitropoulos et al., 2015)

9 This variable comprehends infrastructure like the number of surgery, intensive care rooms, etc. as well as medical equipment such as imaging, diagnosis, sterilization, etc.

10 Note that all the inputs are measured in physical units, no monetary variables have being included in this study.

11 The potential problem that could arise with the inclusion of this variable is that if hospitals are reimbursed according to the number of patient stays, we could expect hospital managers to behave opportunistically in order to increase their hospitals’ revenues.

12 The descriptives for the remaining years are comparable.

13 Although the methodology used in this study to obtain the efficiency values has proved to be robust to outliers, we still run some outlier detection procedures to avoid potential bias in the estimations. We identified nine hospitals that were behaving as outliers, on average.

14 The peer observations are those efficient DMUs with which the inefficient units are directly compared, in order to be fully efficient; that is, they are those reference units that define the efficient production for every inefficient hospital (Coelli et al., 2005; El-Mahgary and Lahdelma, 1995)
