# Supplementary material for: Analyzing the effect of health reforms on the efficiency of Ecuadorian public hospitals
Source: Int J Health Econ Manag. 2023 Mar 16;23(3):361–92. doi: 10.1007/s10754-023-09346-z (PMC10462564; doi:10.1007/s10754-023-09346-z)
Supplement: Supplementary file 1 — (pdf 97 KB) [file 10754_2023_9346_MOESM1_ESM.pdf]

Analyzing the effect of health reforms on the efficiency of Ecuadorian public hospitals

Juan Piedra-Peña[a], Diego Prior[b]
[a]Department of Applied Economics, Universitat Autònoma de Barcelona, Campus Bellaterra, Barcelona, Spain. E-mail: juanandres.piedra@uab.cat.com
[b] Department of Business, Universitat Autònoma de Barcelona, Campus Bellaterra, Barcelona, Spain.

Online Appendix

Table 1 Variable description[a]

Variable	Description
totcam	Total number of hospital beds
m1	Total number of general physicians
m2	Total number of surgeons
m3	Total number of plastic surgeons
m4	Total number of specialized physicians
m5	Total number of resident physicians
m6	Total number of rural physicians
m7	Total number of other physicians
proftit	Health personnel
p1	graduates and technologists
p2	Nursery auxiliary
p3	Administrative personnel
eq1	Stomatology equipment
p4	Stomatology personnel
eq2	Imaging equipment
eq3	Diagnostic equipment
eq4	Treatment equipment
eq5	Physical infrastructure for surgery, obstetrics and intensive care
eq6	Equipment for surgery, obstetrics and intensive care
eq7	Sterilization equipment
eq8	Other equipment

[a] Data from the Annual Survey of Hospital Beds and Discharges 2006–2014.

**Table 2** Technological endowment variables, means and SD.									
	2006	2007	2008	2009	2010	2011	2012	2013	2014
totcam	74.93	74.03	80.88	75.97	82.12	83.10	86.44	87.65	85.04
	(112.93)	(110.68)	(121.41)	(112.10)	(126.47)	(120.47)	(116.18)	(119.57)	(126.61)
m1	3.20	3.18	4.03	4.79	5.40	5.35	7.70	8.39	10.97
	(4.26)	(2.94)	(6.79)	(8.02)	(8.70)	(7.47)	(16.68)	(16.38)	(24.02)
m2	2.43	2.69	2.70	2.76	2.94	3.08	3.21	3.16	3.07
	(2.72)	(3.14)	(2.97)	(2.94)	(3.45)	(3.88)	(4.26)	(4.26)	(4.86)
m3	0.40	0.36	0.39	0.37	0.50	0.40	0.51	0.46	0.39
	(1.00)	(0.93)	(0.97)	(1.00)	(1.32)	(1.18)	(1.43)	(1.13)	(0.97)
m4	18.39	19.13	24.11	24.92	28.08	27.02	29.50	30.96	32.74
	(27.60)	(27.90)	(39.49)	(41.39)	(46.53)	(44.75)	(47.90)	(52.57)	(59.12)
m5	10.14	9.91	9.10	9.73	11.65	14.15	17.24	16.88	19.47
	(19.72)	(18.23)	(15.75)	(14.48)	(17.26)	(22.75)	(28.38)	(25.54)	(32.67)
m6	1.96	1.80	2.05	2.68	2.65	3.01	2.68	3.82	1.05
	(2.53)	(2.39)	(2.39)	(3.81)	(3.42)	(5.19)	(4.83)	(17.10)	(5.33)
m7	1.16	1.41	1.36	1.20	1.64	1.59	1.29	1.62	2.81
	(4.35)	(4.09)	(7.38)	(3.75)	(7.56)	(6.95)	(6.36)	(5.51)	(10.94)
proftit	31.32	32.94	36.42	39.16	48.15	51.12	60.26	63.63	65.53
	(54.41)	(55.59)	(53.99)	(53.66)	(76.70)	(83.66)	(92.52)	(92.00)	(100.16)
p1	3.49	3.43	11.42	12.09	14.95	14.91	18.65	19.63	18.34
	(8.72)	(8.41)	(19.48)	(19.94)	(24.65)	(25.94)	(30.65)	(30.39)	(31.69)
p2	46.14	43.85	47.74	45.78	53.07	50.98	55.20	58.63	56.29
	(81.60)	(69.78)	(81.85)	(81.02)	(91.79)	(89.42)	(97.93)	(101.01)	(98.75)
p3	20.51	20.71	22.88	22.53	25.70	28.85	37.02	35.22	35.29
	(27.22)	(28.38)	(28.87)	(27.08)	(29.98)	(38.43)	(48.45)	(44.86)	(51.46)
p4	3.90	3.86	4.02	3.88	4.22	4.45	4.34	4.50	3.20
	(3.86)	(3.69)	(4.52)	(2.87)	(3.38)	(3.97)	(4.24)	(4.93)	(4.71)
eq1	16.35	17.26	17.63	18.47	19.26	20.33	20.55	46.07	38.43
	(11.00)	(15.52)	(14.48)	(14.96)	(17.01)	(15.90)	(16.47)	(58.31)	(58.46)
eq2	4.60	3.52	3.78	3.83	4.14	4.44	4.62	4.95	5.13
	(16.06)	(3.23)	(3.41)	(3.30)	(4.10)	(4.46)	(4.39)	(5.28)	(5.30)
eq3	4.65	3.53	3.26	3.18	3.91	4.31	4.99	5.19	6.34
	(16.69)	(4.66)	(5.02)	(5.21)	(6.66)	(6.74)	(7.95)	(8.42)	(9.90)
eq4	4.98	6.15	6.71	6.42	7.14	7.54	7.86	8.37	8.21
	(12.49)	(16.95)	(21.29)	(15.76)	(18.41)	(18.59)	(17.40)	(17.06)	(17.63)
eq5	3.63	3.73	4.82	4.72	5.00	5.53	4.95	5.05	4.75
	(3.48)	(3.32)	(5.17)	(4.34)	(4.95)	(8.21)	(6.10)	(5.33)	(4.30)
eq6	29.24	28.29	38.84	44.81	48.09	50.05	55.79	61.10	61.61

Table 2 Technological endowment variables, means and SD.

	2006	2007	2008	2009	2010	2011	2012	2013	2014
	(39.15)	(28.85)	(50.32)	(63.80)	(71.12)	(69.36)	(80.75)	(89.87)	(87.63)
eq7	3.90	3.87	4.08	3.99	4.46	4.44	4.37	4.35	4.25
	(3.21)	(3.06)	(2.93)	(2.90)	(4.93)	(5.11)	(3.21)	(3.25)	(3.42)
eq8	6.13	6.24	4.28	3.97	4.35	4.62	4.83	5.17	4.50
	(7.33)	(7.27)	(3.77)	(2.85)	(2.90)	(3.67)	(3.93)	(3.87)	(2.79)

[a] Data from the Annual Survey of Hospital Beds and Discharges 2006–2014.

[b] Standard deviations in parentheses.
